# Implementation of Nutritional Assessment and Counseling in Physical Therapy Treatment: An Anonymous Cross-Sectional Survey

**DOI:** 10.3390/nu15194204

**Published:** 2023-09-29

**Authors:** Roy Netzer, Michal Elboim-Gabyzon

**Affiliations:** Department of Physical Therapy, School of Social Welfare and Health Sciences, University of Haifa, Haifa 3498838, Israel; netzeroy@gmail.com

**Keywords:** nutrition, assessment, counseling, nutritional knowledge, nutritional lifestyle, physical therapy

## Abstract

In the context of the evolving role of Physical Therapists (PTs) in health promotion, this study explored the incorporation of nutritional assessment and counseling into PTs’ professional practice in Israel. Using an anonymous cross-sectional survey design, the research gauged PTs’ professional background, nutritional knowledge, lifestyle habits, and the extent of nutritional care integration. Our survey gathered data from 409 certified PTs in Israel, revealing inadequate nutritional knowledge, commendable nutritional lifestyles, and limited nutritional care integration. Participants with over 13 years of clinical experience demonstrated significantly higher levels of nutritional assessment and counseling integration within their physical therapy practices. Workplace setting, nutritional lifestyle, and nutritional knowledge emerged as significant predictors for nutritional care integration. Specifically, working in outpatient clinics and possessing better nutritional lifestyles and knowledge were associated with the increased integration of nutritional assessment and counseling within physical therapy practice. These findings underscore the need for targeted interventions and formal nutrition education to bridge the knowledge gaps and optimize patient care. These results advocate for comprehensive nutrition education in physical therapy curricula and the fostering of PTs as role models. Integrating nutrition care could empower PTs to enhance patient outcomes and fulfill their role in preventive healthcare.

## 1. Introduction

The scope of the physical therapy profession has traditionally been perceived as not fully encompassing nutritional assessment and counseling in clinical practice. However, there is a growing recognition of a wellness model in which physical therapists (PTs), similar to other healthcare professionals, play an important role in promoting health, wellness, and preventive healthcare [1]. This shift is exemplified by the American Physical Therapy Association (APTA), which issued a statement affirming the involvement of PTs in health promotion. This includes activities such as screening for inadequate nutrition and providing nutritional guidance within clinical practices [2].

The process of physical therapy rehabilitation, aimed at restoring energy reserves, enhancing body tissues, and improving functionality in patients grappling with acute or chronic illnesses or injuries, necessitates meticulous nutritional considerations [3]. Furthermore, the energy demands induced by physical therapy interventions during treatment underscore the critical importance of upholding a balanced diet. An imbalanced diet could potentially hinder the rehabilitation process, thereby extending the time required to achieve the treatment objectives [3]. In modern practice, PTs are increasingly emphasizing healthy behaviors within clinical approaches, particularly focusing on patients’ physical activity levels while giving comparatively less attention to dietary habits. Challenges hindering the integration of health promotion into physical therapy practice include PTs’ unease about aligning their role in promoting health behavior with patients’ perceptions, as demonstrated by Black et al. (2016) [4]. Notably, a significant majority of outpatient physical therapy clinic attendees (91.3% out of 230) viewed PTs as primarily responsible for guiding their physical activity levels, while fewer believed PTs should advise on fruit and vegetable intake (32.1%). These patients also expected PTs to embody healthy behaviors themselves, encompassing regular physical activity, maintaining a healthy weight, and refraining from smoking [4]. This consensus underscores the role of PTs as health behavior role models, highlighting the potential impact of role modeling in health promotion interventions [5,6].

The concept of the personal scope of practice pertains to the spectrum of activities for which a given physical therapist is educated, trained, and proficient [7]. PTs exhibit distinct expertise in assessing and treating chronic pain, as well as addressing musculoskeletal, neurological, and developmental conditions [7]. Nonetheless, existing research indicates a deficiency in nutritional knowledge among PTs, often stemming from inadequate education regarding their role in delivering nutrition care [7,8,9,10]. Several studies underscore PTs’ willingness to incorporate dietary counseling into their clinical practice [8,9,11,12,13]. Nonetheless, the existing literature lacks a comprehensive comprehension of how nutritional screening and counseling are practically implemented within physical therapy treatments. Specifically, there is a lack of information regarding how the specific professional background characteristics of PTs, such as experience, work setting, and nutritional knowledge, might impact the integration of nutrition-related issues into clinical practice. Moreover, scant insight exists concerning the correlation between PTs’ personal dietary habits and their integration of nutrition-related considerations within clinical practice. Gaining a deeper understanding of these aspects assumes paramount importance for formulating effective strategies aimed at promoting the integration of nutrition-related considerations in the clinical setting.

Therefore, the objectives of this present study are to address the following questions:

1. What professional characteristics within the background of PTs are linked to the inclusion of nutritional assessment and counseling in professional practice?
2. Does a correlation exist between the nutritional knowledge of PTs and the inclusion of nutritional assessment and counseling in professional practice?
3. Is there a relationship between the nutritional lifestyle of PTs and the inclusion of nutritional assessment and counseling in professional practice?

## 2. Materials and Methods

### 2.1. Design

This study utilized an anonymous cross-sectional nationwide online survey.

### 2.2. Participants

#### Eligibility and Exclusion Criteria

Participation was extended to certified PTs holding a bachelor’s degree, with at least one year of clinical experience and actively participating in clinical practice. However, certified PTs possessing an additional degree as registered dietitians were excluded from participation.

### 2.3. Study Procedure

The research protocol followed these distinct steps:

1. Questionnaire Design:

An anonymous survey in Hebrew was formulated by the authors of the study (RN and MEG). The design of the questionnaire was rooted in the “Knowledge and Attitudes of Nutrition, Nursing, Physical Therapy, and Exercise Physiology/Physical Activity & Fitness Majors Regarding Nutrition Questionnaire”, initially developed by Thomas et al. (2006) [14]. Modifications were introduced to tailor the questionnaire to the specific PT population in Israel, incorporating updates from evidence-based nutritional guidelines and the Israel Ministry of Health’s endorsed position paper on optimal nutrition across diverse populations [15,16,17,18].

2. Expert Panel Review:

The survey underwent rigorous assessment by an expert panel comprising 5 certified PTs, 3 registered dietitians, and 3 physical therapy students. Feedback encompassed the questionnaire’s length, clarity, content validity (assessed by dietitians), and alignment with the field of physical therapy. The viewpoints of both PTs and physical therapy students were considered, focusing on the level of question complexity and the pertinence within the context of professional practice.

3. Questionnaire Distribution:

The finalized survey was disseminated to PTs across Israel via “Qualtrics” software [February 2022] (Qualtrics, Provo, UT, USA). The distribution spanned a four-month timeframe (20 March 2022–17 July 2022). Participant recruitment involved a convenience sampling strategy, using social media platforms, physiotherapy professional groups, the newsletter of the Israeli Physiotherapy Society, and word-of-mouth communication within the physical therapy community. The survey included a statement that completing and sending the questionnaire indicated informed consent. This research was approved by the Ethics Committee at the Faculty of Social and Health Sciences, at the University of Haifa (number 2858).

### 2.4. Outcome Measure

The anonymous survey in Hebrew comprised four sections:

1. Personal and professional information—Gathering participants’ data encompassing age, body mass index (BMI), prior nutrition self-counseling, academic background, place of physical therapy studies, workplace, professional experience, and pre-existing nutritional knowledge acquired.
2. Nutrition knowledge test: Comprising 19 questions—1 segment encompassed 7 questions with 3 possible answers (For example, 1 query inquired, “is it recommended to reduce red meat consumption to no more than 300 g per week?” with response options including “yes”, “no”, or “don’t know”), and 12 questions with 5 possible answers (For example, a question explored the range of BMI values indicating risks of morbidity due to overweight or underweight with response options including: “25–29.9”, “20–24.9”, “<18.5 and 30<“, “19–29”, and “Don’t know”).
3. Evaluation of the nutritional lifestyle: Participants responded to 5 statements using a five-point Likert-type scale ranging from 1 (strongly agree) to 5 (strongly disagree) (“I maintain normal body weight to reduce the risk of developing chronic diseases in the present/future”). Additionally, there were 4 questions with 5 possible answers (For example, “How many times a week do you drink alcohol on average?” with response options including: 1. “Rarely”, 2. “1–4 times a month”, 3. “Once a week”, 4. “2–4 times a week”, and 5. “Every day”).
4. Evaluation of nutrition integration in the physical therapy practice: This section consisted of 9 statements, each offering three potential answers: “yes”, “no”, and “sometimes”. For example, “During interview and examination, I ensure that there was no involuntary weight loss exceeding 5%”.

### 2.5. Statistical Analysis

Descriptive statistics were calculated for all outcome measures. For the nutrition knowledge test, means and standard deviations of the correct answers (out of 19) and total scores (out of 100) were calculated for the 19 questions. A higher score indicated higher participant nutritional knowledge. For the evaluation of the nutrition lifestyle score, the overall mean score was calculated using the average of the eight questions. A lower average score suggested a higher level of the nutritional lifestyle of the participants. The evaluation of the nutrition integration in physical therapy practice score was assessed using a total mean score with a range of 9 to 27, where a lower average score indicated a stronger integration of nutrition within physical therapy practice.

The internal consistency of the evaluation of the nutrition lifestyle scale and the evaluation of the nutrition integration scale were determined through Cronbach’s alpha. Alpha values of  ≥0.9 were considered excellent, 0.7  ≤  α  <  0.9 were considered good, 0.6  ≤  α  <  0.7 were acceptable, 0.5  ≤  α  <  0.6 were poor, and α values < 0.5 were considered unacceptable [19].

A bivariate correlation coefficient test was utilized to determine the relationship between the evaluation of the nutrition integration in physical therapy practice mean score variables including age, BMI, nutrition knowledge test mean score, and the evaluation of the nutrition lifestyle mean score.

Differences in means across workplace and years of clinical experience were evaluated using one-way Analysis of Variance (ANOVA) for the total evaluation of the nutrition integration in physical therapy practice mean score. 

A multiple linear regression analysis was conducted. Multiple linear regression analysis was used for predicting nutritional assessment and counseling integration within the physical therapy framework. Variables included professional experience, workplace, age, nutrition knowledge test mean score, and nutrition lifestyle mean score. Statistical significance was set at *p*-values ≤ 0.05.

## 3. Results

### 3.1. Participant Characteristics

The study encompassed a total of 426 respondents. Among these, 4 respondents were excluded from the analysis due to their additional qualifications as registered dietitians, while 13 respondents, whose primary workplace was in academia without clinical practice engagement, were also excluded. Consequently, the study included a final participant count of 409.

### 3.2. Personal and Professional Background Characteristics

Table 1 provides an overview of the background characteristics of the participants. The participants’ mean age was 41.2 ± 10.0 years, with an average BMI of 24.1 ± 3.6. Almost half of the respondents (48.4%) had sought nutritional counseling at least once. Most of the participants received their education in Israel (94.3%) and possessed clinical experience of three years or more (92.6%). A significant portion (63.3%) worked in outpatient clinics. A substantial number of responders indicated a lack of prior nutrition education during their initial degree (66.9%) and other settings (62.8%).

### 3.3. Nutritional Knowledge Level

Detailed scores for the nutrition knowledge assessment consisting of 19 questions are presented in Table 2. The mean total nutrition knowledge score was 43.43 ± 13.75 (out of 100(, with average of 8.25 and standard deviation of 2.61 correct answers out of 19. Analysis revealed that the PTs had a solid understanding of vitamins, minerals, and their roles in normal bodily functions, with 83% accurately identifying essential vitamins for maintaining bone health. Similarly, a substantial number (83%) correctly recognized the recommended BMI index value. Conversely, a majority of PTs (88%) were not aware of the updated food arrangement outlined in the Israel Ministry of Health’s 2020 nutritional recommendations. Gaps in knowledge were observed in areas such as malnutrition, dyslipidemia, and diabetes (laboratory values).

### 3.4. Nutritional Lifestyle

The nutritional lifestyle scale demonstrated acceptable internal consistency (Cronbach alpha = 0.67). Table 3 outlines the total scores for the nutritional lifestyle scale and its eight individual items. With lower scores indicating healthier nutritional lifestyles, PTs demonstrated a favorable nutritional lifestyle with a total score of 18.6 ± 4.6 out of 40.

Most participants rated their lifestyle at a moderate level (2.9 ± 0.9 out of 5) and maintained a healthy body weight (2.1 ± 0.9 out of 5). Additionally, they reported infrequent consumption of fast food (1.8 ± 1.0 out of 5) and a preference for non-processed foods (1.9 ± 0.9 out of 5). However, in contrast to the Ministry of Health’s 2020 position paper, a significant number of PTs consumed animal protein in the form of meat or chicken more than three times a week (3.1 ± 1.4 out of 5).

### 3.5. Nutrition Integration in Physical Therapy Practice

The nutrition integration in physical therapy practice scale exhibited acceptable internal consistency (Cronbach alpha = 0.66). With lower scores indicating higher integration, the mean total score was 20.3 ± 3.2 out of 27, indicating a relatively low integration level of nutrition within PT’s clinical practices. Further details on the individual items are provided in Table 4. Inconsistencies were observed in nutritional questioning during patient interviews (2.0 ± 0.7 out of 3). PTs also did not consistently perform waist circumference tests (2.9 ± 0.3 out of 3) or calculate participants’ BMI indexes (2.9 ± 0.4 out of 3) during clinical examinations. Notably, PTs reported referring patients to clinical dietitians when observing poor nutritional lifestyles (1.5 ± 0.7 out of 3). However, PTs often omitted assessing patient readiness for lifestyle changes and their confidence levels in making such changes (2.1 ± 0.8 out of 3). Additionally, PTs displayed limited engagement in guiding patients with poor nutritional habits toward lifestyle changes and reducing processed food consumption (2.2 ± 0.7 out of 3 and 2.4 ± 0.8 out of 3, respectively).

### 3.6. Nutrition Integration in Physical Therapy Depending on Professional Characteristics

A comparison of the different clinical experience and workplace groups is detailed in Table 5. Participants with over 13 years of clinical experience (F (2, 406) = 4.04, *p* < 0.05) and those working in outpatient clinics (F (3, 403) = 8.6, *p* < 0.001) demonstrated significantly higher levels of nutritional assessment and counseling integration within their physical therapy practices. 

### 3.7. Correlation Analysis

Bivariate correlation coefficient analysis revealed a significant positive low correlation between the mean nutrition integration in physical therapy scores and participants’ mean nutritional lifestyle scores (r = 0.16, *p* < 0.001). A significant negative moderate correlation was observed between the mean nutrition integration score and participants’ mean nutrition knowledge score (r = −0.25, *p* < 0.001). A negative low correlation was observed between the mean nutrition integration score and age (r = −0.12, *p* = 0.01), while no significant correlation was detected with BMI. For further details see Table 6.

### 3.8. Predictors of the Higher Implementation of Nutritional Assessment and Counseling in Physical Therapy Treatment: A Regression Analysis

The results of the multiple regression analysis are presented in Table 7. Workplace setting, nutritional lifestyle, and nutritional knowledge emerged as significant predictors, accounting for 14.89% of the variance (R^2^ = 14.89, F (8, 398) = 8.7, *p* < 0.001). Specifically, working in outpatient clinics and possessing better nutritional lifestyles and knowledge were associated with the increased integration of nutritional assessment and counseling within physical therapy practice.

## 4. Discussion

The present study delves into three fundamental aspects within a domain that has not been deeply examined in prior research: nutritional knowledge, nutritional lifestyle, and the degree of integration of nutritional assessment and counseling within the area of physical therapy practice, with a specific focus on Israeli PTs. The findings revealed that registered PTs in Israel exhibit inadequate nutritional knowledge, commendable nutritional lifestyles, and a low level of integration of nutritional assessment and counseling within their physical therapy practice. In addition, a noteworthy association was demonstrated between enhanced integration and longer clinical experience, along with employment in outpatient clinics.

The study findings revealed considerable knowledge gaps concerning the Ministry of Health’s position paper. Out of eight questions assessing participants knowledge of and familiarity with the 2020 position paper, respondents answered six questions incorrectly. Notably, 88% of respondents offered inaccurate answers concerning the revised food categorizations presented in Israel’s Ministry of Health’s updated dietary guidelines. The current study identified a notable gap in the ability of PTs to recognize and identify crucial health risk factors. A considerable percentage of participants had difficulty correctly identifying markers such as glucose and glycated hemoglobin levels for diabetes diagnosis (60% error rate) and cholesterol levels indicating dyslipidemia (70% error rate), and knowledge gaps were found among PTs in malnutrition detection, with 87% lacking awareness of serum albumin’s importance and 70% being unfamiliar with weight loss percentages as indicators of decline. However, the study’s outcomes emphasize that PTs exhibit a satisfactory understanding of the functions of vitamins and minerals, along with their pivotal role in maintaining proper human body function. The literature underscores PTs’ crucial role in detecting malnutrition risks, especially for those seeking physical therapy [20,21,22,23]. Under 40% of cases receive proper nutritional intervention, emphasizing PTs’ role in recognizing/reporting malnutrition [15,24]. Integration of tools such as food frequency questionnaires and screening tools could substantially enhance PTs’ capability to identify malnutrition risks, ultimately improving patient outcomes and wellbeing [16,25,26]. Further research should assess these tools’ impact on rehabilitation outcomes in physical therapy practice.

A substantial knowledge of nutrition has been substantiated to positively impact the self-efficacy of PTs, leading to enhanced proficiency in dispensing nutritional guidance and managing weight-related concerns [27]. The study by John et al. (2020) also affirming that structured education in nutrition has been influential in facilitating the incorporation of nutrition-related topics into physical therapy practice [12]. Pervious research consistently demonstrates insufficient nutritional knowledge among PTs, evident in studies by Long et al. (1989) and Nelson et al. (1997) [8,10]. Our study’s findings accentuate this trend, revealing even lower nutritional knowledge levels among Israeli PTs. This knowledge gap signifies inadequate nutrition education within physical therapy programs, raising concerns about the relevance and currency of the curriculum content. 

The current findings underscore the necessity for comprehensive nutrition education within entry-level physical therapy programs, a prerequisite already established by educational governing bodies (Commission on Accreditation in Physical Therapy Education—CAPTE) [28]. This skills’ development can be fostered during both undergraduate and graduate physical therapist education programs, supplemented by postdoctoral education or enriched through high-quality evidence-driven continuing education avenues [7].Furthermore, our study’s findings underline the crucial role of nutritional knowledge as a significant predictor for the successful integration of nutritional assessment and counseling within the ambit of physical therapy practice.

The concept of role modeling holds a significant place in shaping healthy behaviors among PTs, as supported by prior research. Studies such as Black et al. (2012) have shown that a substantial number of PTs actively engage in healthy practices, such as regular physical activity, adhering to balanced diets, refraining from smoking, and maintaining healthy weights [5]. This recognition of role modeling’s efficacy underscores the importance of PTs practicing what they advocate, reinforcing their credibility as health educators [5]. In alignment with this trend, our study revealed that registered PTs in Israel exhibit commendable nutritional lifestyles, marked by a healthy body weight maintenance, a preference for plant-based foods, and limited consumption of processed foods and alcohol. Importantly, we uncovered a significant and novel correlation: PTs with healthier nutritional lifestyles tend to integrate nutritional assessment and counseling more effectively into their clinical practice. This observation echoes findings from Frank et al. (2000), which highlighted that physicians with healthy personal habits are more inclined to discuss preventive measures with their patients [29]. These consistent trends underline the potential influence of health professionals’ personal habits on patient interactions, emphasizing the vital role of PTs as role models for positive health behaviors, as demonstrated by Black et al. (2012) [5].

A low level of integration of nutritional assessment and counseling within the area of physical therapy practice was found also in previous studies [1,4,13,27]. Black et al. (2016) investigated patient–PT interactions in outpatient clinics, revealing that while discussions about physical activity were prevalent, weight-related conversations were limited, especially for overweight participants [4]. Similar results were demonstrated by Rea et al. (2004) and O’Donoghue et al. (2014), where PTs emphasized physical activity over other health promotion aspects such as nutrition [1,27]. A recent study by John et al. (2020) examined 51 practicing PTs, exploring their incorporation of nutrition, intentions, and facilitators for integration. In contrast to our findings, many PTs in that study actively discussed nutrition and made referrals to nutrition professionals, most of them working in outpatient clinics. Yet, this research did not delve into outpatient clinics’ role in integration [12].

Our study demonstrated that employment in outpatient clinics emerged as a significant predictor of the heightened integration of nutritional assessment and counseling in clinical practice. Previous studies have documented an array of obstacles that confront PTs in their discussions about nutrition with patients. Among these challenges, time constraints emerge as a recurrent issue, particularly pronounced in dynamic clinical settings characterized by high levels of activity and demand [1,12,30]. Accordingly, it is plausible to posit that PTs working in private clinics might accord more priority to this subject due to the individualized and extended treatment approaches characteristic of this practice setting. These work environments, marked by prolonged treatment durations and extended patient interactions, furnish an environment conducive to the assimilation of nutrition care into practice [7], thereby reinforcing the role of such settings in bolstering nutritional interventions within the domain of physical therapy practice. The majority of survey participants (63%) are employed in outpatient clinics. In private clinics, professionals such as dietitians may not be an integral part of the team, unlike in hospitals and rehabilitation centers. Consequently, physical therapists are expected to acquire foundational knowledge enabling them to provide initial and crucial guidance on nutrition-related matters. This includes tasks such as screening for nutritional deficiencies and referring patients for further consultation and treatment to a qualified dietician [25]. However, the relevance of this argument to non-private outpatient clinics remains uncertain as our questionnaire did not differentiate between clinic types, which calls for further investigation in the future. Future research should also consider exploring the feasibility of integrating nutrition within the demanding clinical contexts of acute or rehabilitation hospitals.

Clinical experience, particularly when 13 years or more, emerged as a notably influential factor, demonstrating higher levels of nutritional assessment and counseling integration within PTs’ physical therapy practice. This observation stands in contrast to the study by John et al. (2020), wherein the significance of years of clinical experience in incorporating nutrition into physical therapy practice was not ascertained, despite a notable proportion of participants in their study having 15 years or more of clinical experience (56.7%) [12]. One possible explanation for this discrepancy lies in the methodological differences in how clinical experience was categorized and measured between the two studies. In our study, clinical experience was grouped into three categories (1–2 years, 3–12 years, above 13 years), whereas John et al. (2020) categorized clinical experience into six groups (0–5, 6–10, 11–15, 16–20, 21–25, 25, 30, 31–35+) [12]. This variation in categorization may have influenced the observed associations between clinical experience and nutrition integration. It is plausible that consolidating the years of experience into larger groups with a larger sample size could potentially yield similar results to our study.

Our study evaluated the integration of nutrition care in physical therapy practices to pinpoint areas needing improvement. Unlike prior research, we quantified the implementation of nutritional diagnosis and counseling, assigning each participant an average score based on recent PT recommendations [1,2,6,7,12,15,16,31]. This revealed specific gaps, such as PTs not urging patients to avoid unhealthy processed foods despite awareness of their harms [32]. This underscores the need for better education to promote healthy lifestyles during PT training. We also identified gaps in PT practices. While weight management guidelines recommend BMI and waist circumference measurement, our study found these are not consistently followed by Israeli PTs [33]. This inconsistency may be due to a lack of training [7,8,9,10,13]. Additionally, incorporating blood test results into clinical assessments and referrals was rare, reflecting the knowledge gap found in this area among participants.

The role of nutrition in the practice of physical therapy should be considered within the framework of each country’s practice act and regulations [15]. For example, Berner et al. (2021) [15] have discussed the legal constraints that impact the practice of physical therapy in the United States, concerning nutrition-related issues. In Israel, the regulation of various health professions, including occupational therapy, physical therapy, communication clinics, and dietetics, was introduced in 2008. According to this law, a qualified physical therapist in Israel holds a valid physical therapist certificate issued by the Ministry of Health [34]. It is worth noting that unlike the American Physical Therapy Association (APTA) [2], which explicitly defines the physical therapist’s role in promoting health, including involvement in nutrition, and has issued a statement confirming their participation in health promotion, professional organizations in Israel have not addressed the role of physical therapists in nutrition. In Israel, only individuals possessing a Nutrition-Dietetics Certificate from the Ministry of Health can officially identify themselves as nutritionists-dietitians [35]. There is no legal prohibition preventing health professionals such as physical therapists from discussing nutritional recommendations with patients, as long as they refrain from identifying themselves as “nutritionists” or “dietitians” [35]. Accordingly, a physical therapist can offer primary dietary recommendations and screen for potential nutritional impairment. However, when required, they must refer the patient to qualified dietitians for further consultation, assessment, and treatment. It is worth emphasizing that the primary objective of incorporating nutritional knowledge into physical therapy studies is to provide comprehensive and professional care to patients as part of a multidisciplinary team. Furthermore, there is an urgent imperative to establish a multidisciplinary committee involving national professional organizations in physical therapy and nutrition. Such a committee would play a crucial role in clearly defining the roles of each profession and fostering interprofessional cooperation to enhance individual health.

Addressing these gaps necessitates formal education to empower PTs to confidently integrate nutrition care into practice [7,12,13,28,33]. Strategies proposed by experts, such as assessing behavior change readiness and setting S.M.A.R.T. goals, can enhance patient-centered nutritional care delivery [16,36]. Reinforcing the APTA’s emphasis on detailed nutritional recommendations can further enhance PTs’ roles as health advocates, positively impacting patient outcomes and promoting overall wellbeing [2].

This study was accompanied by several limitations warranting consideration. The utilization of a cross-sectional design within this investigation hinders the establishment of causal relationships among the variables under scrutiny. While this approach permits the observation of associations, it falls short in ascertaining the directionality of these relationships. Furthermore, the reliance upon self-report measures, although enriching in terms of the information garnered, introduces potential biases such as recall bias and social desirability bias. In conclusion, this study, while navigating several limitations, sets the stage for future research endeavors to address these constraints.

## 5. Conclusions

PTs in Israel displayed inadequate nutritional knowledge and a limited incorporation of nutritional assessment and counseling within their clinical practice. Yet, PTs demonstrated commendable nutritional lifestyles. Greater clinical experience demonstrated significantly higher levels of nutritional integration. Workplace setting, nutritional lifestyle, and nutritional knowledge emerged as significant predictors for nutritional care integration. Our findings also point to discrepancies, suggesting a need for targeted interventions and formal education to enable effective nutrition care integration. Future research could explore nutrition education in physical therapy curricula and assess the impact of specific nutritional interventions and evaluate the utility of nutritional screening tools and standardized questionnaires in optimizing patient care within physical therapy rehabilitation.

## Figures and Tables

**Table 1:** Background Characteristics.

Characteristics		Participants (*n* = 409)
Age, years, mean (SD)		41.2 (10.0)
BMI, mean (SD)		24.1 (3.6)
Professional seniority, year *n* (%)	1–2	30 (7.3)
	3–12	191 (46.7)
	13 and above	188 (45.9)
Additional degree/student for an additional degree other than a bachelor’s degree in physical therapy? Yes, *n* (%)	Bachelor’s degree (not in physical therapy)	23 (5.6)
	Master’s degree (in physical therapy/not in physical therapy)	121 (29.5)
	Doctorate (in physical therapy/not in physical therapy)	5 (1.2)
	No	260 (63.5)
Location of the University of the entire PT program? *n* (%)	Israel	386 (94.3)
	Abroad	23 (5.6)
Main workplace set *n* (%) *	Outpatient clinics	258 (63.3)
	Acute Hospitals	48 (11.7)
	Rehabilitation hospitals/Nursing homes	48 (11.7)
	Child development	53 (13.0)
Prior referral/application for nutritional counseling *n* (%)	Yes	198 (48.4)
	No	211 (51.5)
Acquired knowledge in nutrition during the degree? *n* (%)	Yes	73 (17.8)
	No	274 (66.9)
	Don’t remember	62 (15.1)
Acquired knowledge of nutrition in other settings? *n* (%)	Yes	152 (37.1)
	No	257 (62.8)

* Two missing.

**Table 2:** Nutrition Knowledge Questionnaire.

Item		Frequency (Percent)
(1) What is the arrangement of food according to the position paper on the nutritional recommendations of the Israel Ministry of Health in 2020?	1. Pyramid	194 (47.4)
	2. Table	3 (0.7)
	3. Pai	45 (11)
	4. Rainbow *	50 (12.2)
	5. Don’t know	117 (28.6)
The following questions (2–8) refer to the nutritional recommendations according to the position paper of the Ministry of Health. Please mark true/false/don’t know:		
(2) It is recommended to eat lean chicken/turkey every day:	1. True	104 (25.4)
	2. False *	162 (39.6)
	3. Don’t know	143 (34.9)
(3) It is recommended to minimize the consumption of red meat/beef to consume no more than 300 g per week	1. True *	318 (77.7)
	2. False	19 (4.6)
	3. Don’t know	72 (17.6)
(4) Industrial foods (ketchup, snacks, processed meat) are allowed for consumption in the amount of 2–3 portions per week	1. True *	81 (19.8)
	2. False	171 (41.8)
	3. Don’t know	157 (38.3)
(5) It is advisable to reduce alcohol consumption to zero as much as possible	1. True *	151 (36.9)
	2. False	145 (35.4)
	3. Don’t know	113 (27.6)
(6) The arrangement of foods recommended by the Ministry of Health is based on the consumption of unprocessed (natural) food, mainly from plants	1. True *	218 (53.3)
	2. False	65 (15.8)
	3. Don’t know	126 (30.8)
(7) The recommendation for proper nutrition according to the Ministry of Health is based on the recommended frequency of consumption	1. True	166 (40.5)
	2. False *	42 (10.2)
	3. Don’t know	201 (49.1)
(8) The recommendation for proper nutrition according to the Ministry of Health does not refer to the degree of processing of the food	1. True	51 (12.4)
	2. False *	196 (47.9)
	3. Don’t know	162 (39.6)
(9) What is the role of calcium in the body?	1. An essential mineral for muscle contraction *	322 (78.7)
	2. Essential for human fertility	0 (0)
	3. An essential mineral for the synthesis of red blood cells	18 (4.4)
	4. Helps keep blood sugar in the normal range	3 (0.7)
	5. Don’t know	66 (16.1)
(10) Which mineral is important for maintaining a healthy immune system?	1. Zinc *	152 (37.1)
	2. Calcium	8 (1.9)
	3. Potassium	8 (1.9)
	4. Iron	72 (17.6)
	5. Don’t know	169 (41.3)
(11) Which mineral may cause abnormal heart function in a situation of excessive consumption?	1. Chromium	4 (0.9)
	2. Iron	3 (0.7)
	3. Potassium *	314 (76.7)
	4. Zinc	9 (2.2)
	5. Don’t know	79 (19.3)
(12) Which main vitamins are essential for maintaining normal bone mass?	1. Vitamin A, E and Calcium	19 (4.6)
	2. Vitamin A, B and Calcium	18 (4.4)
	3. Vitamin D, K and Calcium *	340 (83.1)
	4. Iron, Folic acid and Hemoglobin	2 (0.4)
	5. Don’t know	30 (7.3)
(13) Which population will benefit from the benefits of consuming protein beyond the dietary recommendations of 0.8–1 g of protein per kg of body weight for the general population?	1. Old people suffering from sarcopenia *	304 (74.3)
	2. Chronic kidney failure patients	12 (2.9)
	3. Young and healthy untrained population	14 (3.4)
	4. Patients suffering from irritable bowel syndrome	1 (0.2)
	5. Don’t know	78 (19)
(14) What percentage of unplanned weight loss in a period of 3–6 months constitutes a marker that patients are suspected of nutritional deterioration and should be referred to a specialist?	1. Sudden weight loss of 2.5%	20 (4.8)
	2. Sudden weight loss of 5% *	126 (30.8)
	3. Sudden weight loss of 1.8%	7 (1.7)
	4. Sudden weight loss of 3.5%	16 (3.9)
	5. Don’t know	240 (58.6)
(15) What is the serum albumin index that signals that patient are suspected of being malnourished and/or are receiving a diet with insufficient protein and energy levels and should be referred to a specialist?	1. Less than 5 g/dL	12 (2.9)
	2. Less than 7.5 g/dL	4 (0.9)
	3. Less than 4 g/dL	12 (2.9)
	4. Less than 3.5 g/dL *	54 (13.2)
	5. Don’t know	327 (79.9)
(16) What are fasting glucose and glycated hemoglobin levels that indicate a diagnosis of diabetes and require a doctor’s visit?	1. Over 126 mg of glucose and 6.5% glycated hemoglobin *	167 (40.8)
	2. Over 100 mg of glucose and 5% glycated hemoglobin	113 (27.6)
	3. Over 80 mg of glucose and 2.5% glycated hemoglobin	4 (0.9)
	4. Over 115 mg of glucose and 5.2% glycated hemoglobin	44 (10.7)
	5. Don’t know	81 (19.8)
(17) What are the cholesterol levels that indicate that patients are in dyslipidemia (disorder in the metabolism of blood lipids) and need a referral to a nutritional change as part of multi-professional rehabilitation in order to maintain health?	1.HDL < 80, LDL > 70, Total cholesterol > 100	26 (6.3)
	2. HDL < 60, LDL > 100, Total cholesterol > 150	38 (9.2)
	3. HDL < 40, LDL > 130, Total cholesterol > 200 *	121 (29.5)
	4. HDL < 50, LDL > 120, Total cholesterol > 170	20 (4.8)
	5. Don’t know	204 (49.8)
(18) What is the range of values that indicates a danger of morbidity due to overweight/underweight according to the BMI index?	1. Between 25–29.9	31 (7.5)
	2. Between 20–24.9	4 (0.9)
	3. From 30 and above, and from 18.5 and below *	341 (83.3)
	4. Between 19–29	3 (0.7)
	5. Don’t know	30 (7.3)
(19) What are the identifiable characteristics in patients that indicate that they are at risk of malnutrition and should be referred for further dietary treatment?	1. Baldness, profuse sweating, accelerated heart rate at rest, profuse secretions, decreased sleep quality and decreased libido	20 (4.8)
	2. Functional decline, decrease in muscle mass, loss of subcutaneous fat, unplanned weight loss and insufficient energy supply through food *	273 (66.7)
	3. Decreased libido, poor appetite, fertility problems, poor sleep, diarrhea, and constipation	16 (3.9)
	4. Lack of motivation to engage in physical activity, depression, lack of proportion between height and weight, excessive sleep, and low caloric expenditure	14 (3.4)
	5. Don’t know	86 (21.0)

* The correct answer.

**Table 3:** Nutritional Lifestyle.

Variable	Mean (SD)
Evaluation of Nutrition Lifestyle (The lower the score, the higher the nutritional lifestyle of the participants) [Range: 1–5]	
1. How many times a week do you eat fast food outside the house or order fast food?	1.8 (1.0)
2. How many times a week do you drink alcohol on average?	2.1 (1.1)
3. How would you rate your nutritional lifestyle?	2.9 (0.9)
4. I tend to minimize the consumption of processed/ultra-processed foods and prefer non-processed foods.	1.9 (0.9)
5. As part of my food selection process I tend to look at its ingredients, nutritional components, and whether it carries a Ministry of Health red/green label.	2.2 (1.0)
6. I tend to prefer plant-based foods in my daily consumption, making sure to diversify my daily consumption of legumes, fruits and vegetables, nuts, and vegetable oils.	2.3 (1.0)
7. I consume animal protein in the form of meat/chicken more than 3 times a week	3.1 (1.4)
8. I maintain normal body weight to reduce the risk of developing chronic diseases in the present/future	2.1 (0.9)
Nutrition Lifestyle mean score [Range: 8–40]	18.6 (4.6)

**Table 4:** Implementation of nutritional assessment and counseling in physical therapy treatment.

Variable	Mean (SD)
Evaluation of nutrition integration in the physical therapy practice (The lower the average score, the higher the nutritional lifestyle of the participants) [Range: 1–3]	
1. During an interview with patients, I ask them about their lifestyle, including their dietary habits	2.0 (0.7)
2. During an interview with patients with poor nutritional habits, I examine their readiness to change their lifestyle and the degree of confidence they have in their ability to do so	2.1 (0.8)
3. During the clinical examination I conduct a waist circumference test as a risk factor for morbidity.	2.9 (0.3)
4. During the clinical examination, I take weight and height values to assess the patient’s BMI and use this index as a risk factor for morbidity.	2.9 (0.4)
5. During the interview and the clinical examination, I make sure that there was no involuntary weight loss of more than 5%.	1.8 (0.9)
6. When accepting new patients, I examine current blood tests and use them in the clinical assessment/referral to a specialist. (Anemia due to nutritional deficiency in vitamins/minerals, lack of albumin, cholesterol/fats/glucose level, etc.)	2.4 (0.7)
7. When I notice a poor nutritional lifestyle, I guide the patients to change their nutritional lifestyle in accordance with the latest nutritional recommendations of the Ministry of Health in order to improve their health.	2.2 (0.7)
8. I encourage patients to stop consuming processed/ultra-processed foods and prefer non-processed foods	2.4 (0.8)
9. I refer patients who need nutritional advice and diagnosis to a clinical dietitian.	1.5 (0.7)
Nutrition integration in the physical therapy practice mean score [Range: 9–27]	20.3 (3.2)

**Table 5:** Comparative analysis of background characteristics and the total implementation of nutritional assessment and counseling score.

Measure		Total Nutrition Integration in Physical Therapy Practice Scale Score	Test Statistic	*p*-Value
		Mean (Standard Deviation)		
Professional seniority	1–2 Years	21.4 (3.1)	F (2, 406) = 4.04	<0.05
	3–12 Years	20.6 (3.3)		
	13 Years and above	19.9 (3.1)		
Main workplace	1. Outpatient clinics	19.8 (3.1)	F (3, 403) = 8.6	<0.0001
	2. Acute Hospitals	21.4 (3.5)		
	3. Rehabilitation hospitals/Nursing homes	22.0 (2.6)		
	4. Child development	20.5 (3.6)		

**Table 6:** Bivariate correlation coefficient tests between the total nutrition integration in physical therapy practice scale score and the total nutritional lifestyle score, age, BMI, and total nutrition knowledge score.

Variable	Total Nutrition Integration in Physical Therapy Practice Scale Score	
	Mean (SD)	r (*p*-Value)
Age (years) ^a^	41.2 (10.0)	−0.12 (0.01)
BMI ^a^	24.1 (3.6)	−0.05 (0.28)
Total nutritional lifestyle score ^a^	18.6 (4.6)	0.16 (<0.001)
Total nutrition knowledge score ^a^	43.4 (13.7)/8.25 (2.6)	−0.26 (<0.0001)

^a^ Pearson correlation coefficient.

**Table 7:** Regression analysis of variable predictors for higher implementation of nutritional assessment and counseling in physiotherapy treatment.

Variable	Total Nutrition Integration in Physical Therapy Practice Scale Score			
	Parameter Estimate	Standard Error	Standardized Estimate	T Value (*p* Value)
Professional seniority—1–2 years vs. over 13 years	0.91	0.78	0.07	1.1 (0.24)
Professional seniority—3–12 years vs. over 13 years	0.45	0.49	0.07	0.92 (0.36)
Workplace—acute hospital vs. outpatient clinic	1.41	0.48	0.14	2.96 (<0.01)
Workplace—rehabilitation hospital/nursing home vs. outpatient clinic	1.91	0.48	0.19	3.99 (<0.0001)
Workplace—child development vs. outpatient clinic	0.71	0.45	0.07	1.57 (0.11)
Age	−0.005	0.02	−0.01	−0.22 (0.82)
Total nutritional lifestyle score	0.08	0.03	0.11	2.36 (<0.05)
Total nutrition knowledge score	−0.05	0.01	−0.24	−5.17 (0.0001)
F (8, 398) = 8.7, *p* < 0.0001, R^2^ = 14.89 **				

** Two missing values.

## Data Availability

Data available on request due to restrictions, e.g., privacy or ethical.
